# Supplementary material for: Association of Toll-Like Receptor Gene Polymorphisms with Tuberculosis in HIV-Positive Participants
Source: Epigenomes. 2023 Jul 25;7(3):15. doi: 10.3390/epigenomes7030015 (PMC10443360; doi:10.3390/epigenomes7030015)
Supplement: Supplementary file 1 [file epigenomes-07-00015-s001.zip › epigenomes-2453853-supplementary.pdf]

Supplementary Material

Association of Toll-Like Receptor Gene Polymorphisms with Tuberculosis in HIV-Positive Participants

Svetlana Salamaikina [1,*], Vitaly Korchagin [1], Ekaterina Kulabukhova [1,2], Konstantin Mironov [1], Vera Zimina [2], Alexey Kravtchenko [1] and Vasily Akimkin [1]

[1] Central Research Institute of Epidemiology Federal Service for Surveillance on Consumer Rights Protection and Human Wellbeing Russian Federation, Novogireevskaya Str. 3a, 111123 Moscow, Russia

[2] Medical Institute, The Peoples' Friendship University of Russia (RUDN University), Miklukho-Maklaya Str. 6, 117198 Moscow, Russia

* Correspondence: salamaikina.sa@phystech.edu

Supplementary Table S1. Oligonucleotide characteristics.

Gene, SNP	Name	5'–3' sequence
TLR1, rs5743551	TLR1-F	AAT CCA GGC TGA GGG AGC AA
	TLR1-R	ACT GGG AGT AGT GGG GAT GAG T
	TLR1-A	(FAM)GCTGAGAAGCTTCC(BHQ1)[1]
	TLR1-G	(R6G)GCTGAGGAGCTTCC(BHQ1)[1]
TLR2, rs5743708	TLR2-F	CATTCTTCTGGAGCCCATTGAGA
	TLR2-R	CGCAGCTCTCAGATTTACCCAA
	TLR2-G	(FAM)GCTGCGGAAGAT(BHQ1)[1]
	TLR2-A	(R6G)AAGCTGCAGAAGAT(BHQ1)[1]
TLR2, rs3804100	TLR2-F1	CTGAAACTTGTCAGTGGCCAGAA
	TLR2-R1	TTCCAGTGTCTTGGGAATGCA
	TLR2-C	(FAM)TACACAGCGTAACAG(BHQ1)[1]
	TLR2-T	(R6G)TACACAGTGTAACAG(BHQ-1)[1]
TLR4, rs4986790	TLR4-F	GGCCTGTGCAATTTGACCATT
	TLR4-R	GTCACACTCACCAGGGAAAATGA
	TLR4-A	(FAM)CCTCGATGATATTATTG(BHQ1)[1]
	TLR4-G	(R6G)CCTCGATGGTATTATTG(BHQ1)[1]
TLR6, rs5743810	TLR6-F	CTATGTGGTTGAGGGTAAAATTCAGTAAG
	TLR6-R	TGAATGATGACAACTGTCAAGTTTTC
	TLR6-A	(FAM)AAGGTTGAACCTCTG(BHQ1)[1]
	TLR6-G	(R6G)AGGTTGGACCTCTG(BHQ1)[1]
TLR8, rs3764880	TLR8-F	GCTGCTGCAAGTTACGGAATGAA
	TLR8-R	GGGTCAGAAACCCCATATTCTGTT
	TLR8-A	(FAM)CAGAAACATGGTAAG(BHQ1)[1]
	TLR8-G	(R6G)CAGAAACGTGGTAAG(BHQ1)[1]

[1] The probes contain from 4 to 7 locked nucleic acids

Supplementary Table S2. Association of studied TLR gene polymorphisms genotypes with the risk of tuberculosis coinfection in HIV-positive individuals.

SNP	HIV	Allele 1 freq	HIV+TB	Allele 2 freq	OR CI95%	*p*
rs4986790 (*TLR4*)						
Codominant						
A/A	207	84.1	223	91.4	1.00	0.02092
A/G	37	15.0	21	8.6	0.53(0.30-0.93)	
G/G	2	0.8	0	0.0		
Dominant						
A/A	207	84.1	223	91.4	1.00	0.01374
A/G-G/G	39	15.9	21	8.6	0.50(0.28-0.88)	
Recessive						
A/A-A/G	244	99.2	244	100.0	1.00	0.499
G/G	2	0.8	0	0.0		
Overdominant						
A/A-G/G	209	85.0	223	91.4	1.00	0.02659
A/G	37	15.0	21	8.6	0.53(0.30-0.94)	
log-Additive						
0,1,2	246	50.2	244	49.8	0.49(0.29-0.85)	0.02092
rs5743708 (*TLR2*)						
Codominant						
G/G	237	96.3	230	94.3	1.00	0.3331
G/A	9	3.7	13	5.3	1.49(0.62-3.55)	
A/A	0	0.0	1	0.4		
Dominant						
G/G	237	96.3	230	94.3	1.00	0.2748

G/A-A/A	9	3.7	14	5.7	1.60(0.68-3.78)	
Recessive						
G/G-G/A	246	100.0	243	99.6	1.00	0.498
A/A	0	0.0	1	0.4		
Overdominant						
G/G-A/A	237	96.3	231	94.7	1.00	0.3711
G/A	9	3.7	13	5.3	1.48(0.62-3.53)	
log-Additive						
0,1,2	246	50.2	244	49.8	1.66(0.73-3.75)	0.3331
rs5743551 (*TLR1*)						
Codominant						
A/A	117	47.6	96	39.3	1.00	0.1808
A/G	97	39.4	113	46.3	1.42(0.97-2.08)	
G/G	32	13.0	35	14.3	1.33(0.77-2.31)	
Dominant						
A/A	117	47.6	96	39.3	1.00	0.0664
A/G-G/G	129	52.4	148	60.7	1.40(0.98-2.00)	
Recessive						
A/A-A/G	214	87.0	209	85.7	1.00	0.6668
G/G	32	13.0	35	14.3	1.12(0.67-1.88)	
Overdominant						
A/A-G/G	149	60.6	131	53.7	1.00	0.1237
A/G	97	39.4	113	46.3	1.33(0.93-1.90)	
log-Additive						
0,1,2	246	50.2	244	49.8	1.22(0.94-1.58)	0.1277
rs3804100 (*TLR2*)						
Codominant						

T/T	193	78.5	190	77.9	1.00	0.7365
C/T	48	19.5	51	20.9	1.08(0.69-1.68)	
C/C	5	2.0	3	1.2	0.61(0.14-2.59)	
Dominant						
T/T	193	78.5	190	77.9	1.00	0.8752
C/T-C/C	53	21.5	54	22.1	1.03(0.67-1.59)	
Recessive						
T/T-C/T	241	98.0	241	98.8	1.00	0.4807
C/C	5	2.0	3	1.2	0.60(0.14-2.54)	
Overdominant						
T/T-C/C	198	80.5	193	79.1	1.00	0.7017
C/T	48	19.5	51	20.9	1.09(0.70-1.69)	
log-Additive						
0,1,2	246	50.2	244	49.8	0.99(0.67-1.45)	0.9585
rs5743810 (*TLR6*)						
Codominant						
G/G	150	61.0	163	66.8	1.00	0.0522
A/G	76	30.9	73	29.9	0.88(0.60-1.31)	
A/A	20	8.1	8	3.3	0.37(0.16-0.86)	
Dominant						
G/G	150	61.0	163	66.8	1.00	0.1792
A/G-A/A	96	39.0	81	33.2	0.78(0.54-1.12)	
Recessive						
G/G-A/G	226	91.9	236	96.7	1.00	0.01878
A/A	20	8.1	8	3.3	0.38(0.17-0.89)	
Overdominant						
G/G-A/A	170	69.1	171	70.1	1.00	0.8143

A/G	76	30.9	73	29.9	0.95(0.65-1.40)	
log-Additive						
0,1,2	246	50.2	244	49.8	0.74(0.55-1.00)	0.04759
rs3764880 (*TLR8*)						
Dominant						
A/A	151	61.4	140	57.4	1.00	0.3667
A/G-G/G	95	38.6	104	42.6	1.18(0.82-1.69)	
